# Diplopia and bilateral optic disc swelling as the initial presentation of B-lymphoblastic lymphoma: a case report

**DOI:** 10.3389/fonc.2026.1763049

**Published:** 2026-03-17

**Authors:** Cheng-Han Chang, Yu-Hung Wang, Bor-Sheng Ko, Yi-Hsuan Wei

**Affiliations:** 1 College of Medicine, National Taiwan University, Taipei, Taiwan; 2 Department of Hematological Oncology, National Taiwan University Cancer Center, Taipei, Taiwan; 3 Division of Hematology, Department of Internal Medicine, National Taiwan University Hospital, Taipei, Taiwan; 4 School of Medicine, National Taiwan University College of Medicine, Taipei, Taiwan; 5 Department of Ophthalmology, National Taiwan University Hospital, Taipei, Taiwan

**Keywords:** B-lymphoblastic lymphoma (B-LBL), diplopia, intracranial hypertension, papilledema, Philadelphia chromosome

## Abstract

B-lymphoblastic lymphoma (B-LBL) is an uncommon and aggressive hematologic malignancy that rarely presents with primary ocular manifestations. Initial presentation with ocular or neuro-ophthalmic symptoms is particularly unusual and may mimic more common intracranial tumors. We report a 21-year-old man with a one-year history of intermittent headaches, initially managed as migraine. He developed diplopia over one month and was referred to ophthalmology, where examination revealed left-sided proptosis and bilateral optic disc swelling with hemorrhage. Neuroimaging demonstrated a large invasive 9 cm extra-axial mass involving the sphenoid wing, orbit, and frontotemporal region, with ventricular compression and early brain herniation, initially suggestive of a primary brain tumor. CT-guided biopsy confirmed B-LBL. Bone marrow examination revealed focal lymphoma involvement, while positron emission tomography (PET) revealed systemic disease with osseous lesions involving the cervical and thoracic vertebrae and left scapula. Cytogenetic analysis of bone marrow confirmed the presence of the Philadelphia chromosome, while real-time RT-PCR detected the BCR-ABL1 fusion transcripts. Subsequently, he was started on systemic therapy with dasatinib in combination with the HyperCVAD chemotherapy regimen. This case illustrates that B-LBL may masquerade as a large intracranial mass, presenting initially with optic disc swelling due to intracranial hypertension. Ocular features such as diplopia in the setting of chronic unexplained headache should prompt careful fundus examination, as it may reveal critical clues to underlying intracranial pathology. Early neuroimaging, timely biopsy, and rapid initiation of systemic therapy remain critical for optimizing outcomes.

## Introduction

B-lymphoblastic lymphoma (B-LBL) is an uncommon and aggressive neoplasm of immature B cells, accounting for fewer than 2% of all non-Hodgkin lymphomas and approximately 10% of lymphoblastic lymphomas (1). B-LBL and B-cell acute lymphoblastic leukemia both originate from the precursor B-cell stage, so they are considered a spectrum of the same disease entity, i.e. precursor B-cell neoplasm. Per the World Health Organization (WHO) classification, B-LBL can be distinguished from B-ALL by having marrow blasts less than 25% (2). It typically presents as extranodal masses involving the skin, bone, or mediastinum, manifesting with localized swelling, pain, or compressive symptoms. In contrast, primary ocular or orbital manifestations are exceptionally rare (3). Central nervous system (CNS) involvement occurs in fewer than 5% of cases and usually develops during advanced disease or relapse rather than at presentation (1). When present, CNS involvement may manifest with signs of elevated intracranial pressure, such as headache, nausea, vomiting, and papilledema, or with focal neurological deficits, including diplopia, cranial nerve palsies, seizures, or altered mental status (4, 5). Because of this rarity, patients with ophthalmic or neuro-ophthalmic symptoms are often initially misdiagnosed with more common conditions, leading to delays in diagnosis and treatment.

The present case is unique in that the patient initially presented with diplopia, unilateral proptosis due to orbital extension of the mass, and bilateral optic disc swelling secondary to intracranial hypertension. However, further evaluation revealed a large invasive extra-axial mass, and histopathology confirmed B-LBL with bone marrow involvement.

This case highlights the importance of considering hematologic malignancies in the differential diagnosis of unexplained optic disc edema and diplopia, particularly in young patients. Recognition of such atypical presentations is critical for early systemic evaluation and timely initiation of appropriate therapy (4–6).

## Case presentation

**Figure 1:**
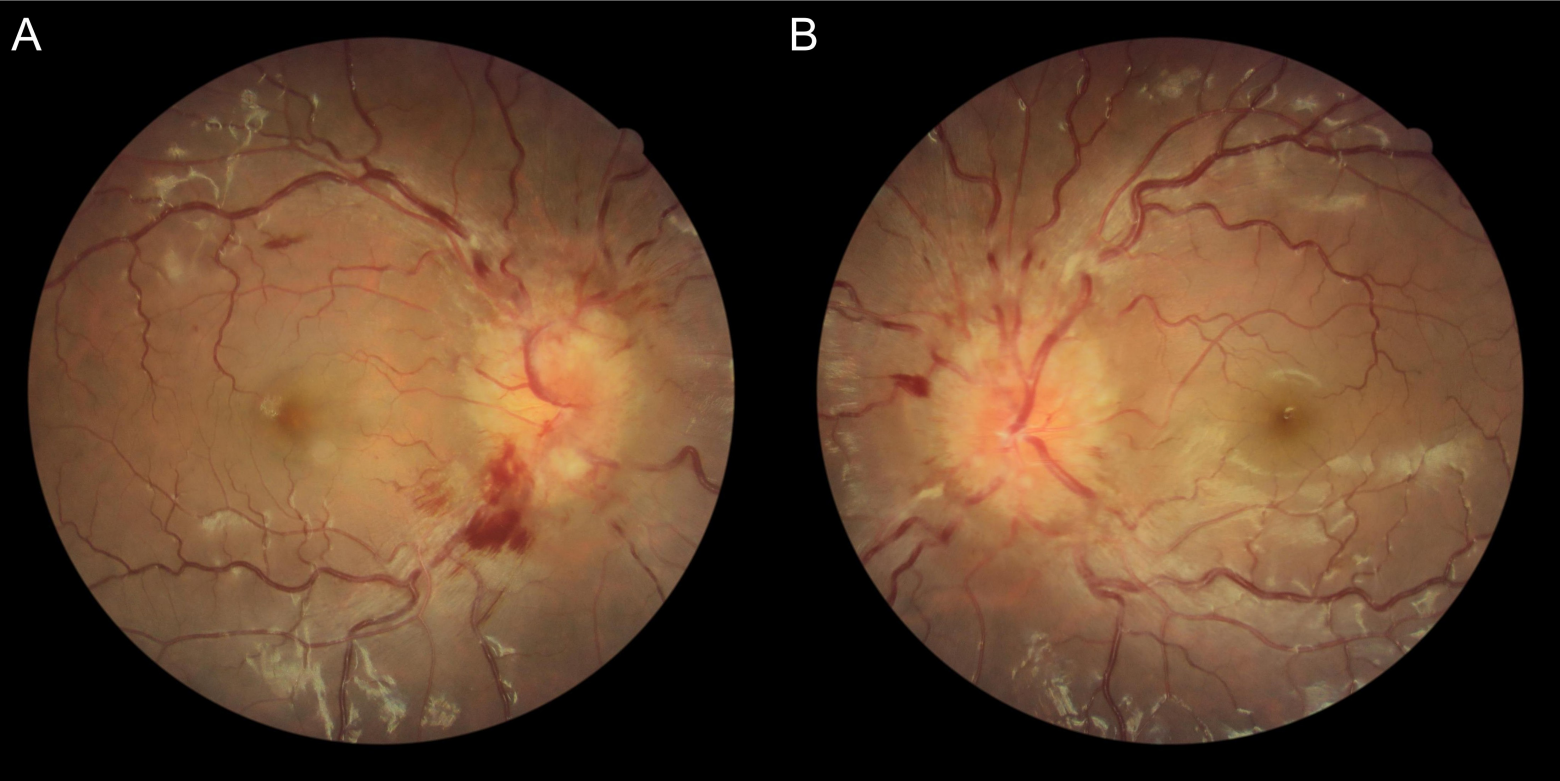
Color fundus photography. [**(A)** OD; **(B)** OS] Fundus photographs at initial presentation demonstrated bilateral optic disc swelling with peripapillary hemorrhages, consistent with papilledema caused by increased intracranial pressure.

A 21-year-old previously healthy man, without significant family or psychosocial history, began to experience a one-year history of intermittent headache, which was treated as migraine with propranolol prophylaxis. However, he then developed a one-month history of binocular diplopia that resolved with occlusion of either eye. He presented to the outpatient clinic with ophthalmologic findings of elevated intraocular pressures of 27.8 mmHg in the right eye (OD) and 29.7 mmHg in the left eye (OS), best corrected visual acuity of 0.8 (20/25) OD and 0.7 (20/30) OS. Ocular alignment testing revealed left hypotropia and exotropia on Hirschberg examination. Extraocular motility demonstrated limitation of upward, medial, and lateral movement in the left eye, suggestive of likely multiple cranial neuropathies, including partial third nerve and sixth nerve palsies. Mild left-sided ptosis was noted. Fundus photography revealed bilateral optic disc swelling with peripapillary hemorrhages (**Figure 1**). Hertel exophthalmometry showed 14 mm OD and 20 mm OS, consistent with left eye proptosis. The anterior segment examination was otherwise unremarkable. Based on the combination of bilateral optic disc swelling, cranial nerve involvement, and chronic headache, increased intracranial pressure (ICP) was suspected, and he was admitted for urgent evaluation.

**Figure 2:**
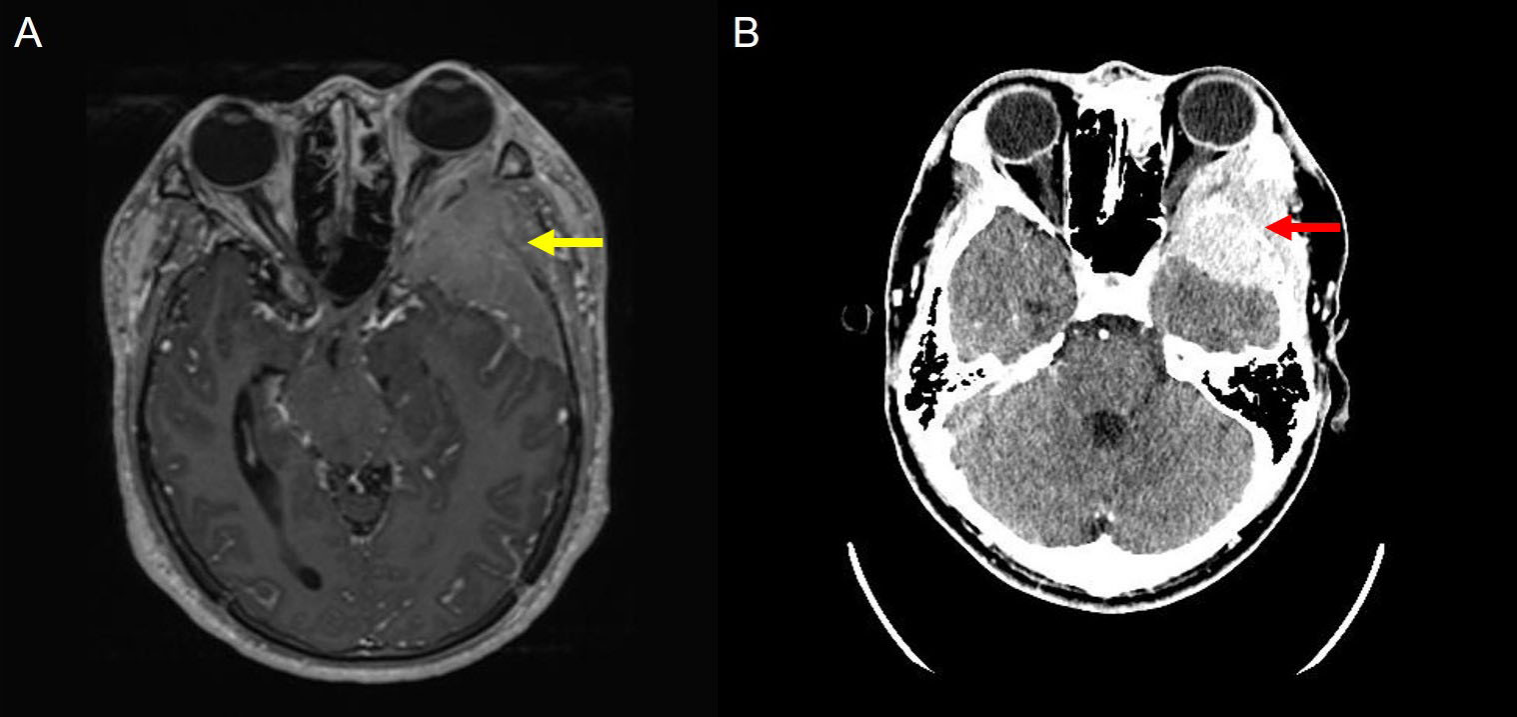
Magnetic resonance imaging (MRI) and computed tomography (CT). **(A)** MRI Axial post-contrast T1-weighted image demonstrates a large extra-axial enhancing mass centered at the left sphenoid wing (yellow arrow), extending into the left orbit and frontotemporal convexity, with significant mass effect. **(B)** CT Axial view showing an ill-defined, heterogeneous soft tissue mass involving the left sphenoid wing and extending into the left frontotemporal region and orbital cavity (red arrow).

Initial neuroimaging demonstrated a large 9 cm mass involving the left sphenoid wing, frontotemporal skull, cortical surface, orbital cavity, and subscalp region, with encasement of adjacent cortical branches of the left middle cerebral artery (M2–M3 segments) and associated mass effect, including ventricular compression and early uncal herniation (**Figure 2A**). Computed tomography confirmed a large osteolytic soft tissue mass in the left lateral sphenoid extending to the frontotemporal region (**Figure 2B**). Due to radiological evidence of impending herniation, lumbar puncture was initially contraindicated. He was started on intravenous mannitol and high-dose methylprednisolone for cerebral edema.

Subsequent CT-guided biopsy revealed atypical lymphoid cells positive for CD79a, CD34, TdT, CD43, PAX5, and CD19, and negative for CK, CD45, CD138, EBER, MPO, CD3, synaptophysin, chromogranin, and ALK, with a Ki-67 proliferation index of 65%, confirming the diagnosis of B-lymphoblastic lymphoma (B-LBL).

Bone marrow examination showed hypocellularity with 65% hematopoietic elements and a myeloid-to-erythroid ratio of 3:1. Focal replacement by blastoid cells was observed, which were immunohistochemically positive for CD19, TdT, and CD34, but negative for CD3 and MPO. Flow cytometry demonstrated 4.27% of cells within the blast region with an immunophenotype consistent with precursor B-LBL, confirming marrow involvement.

**Figure 3:**
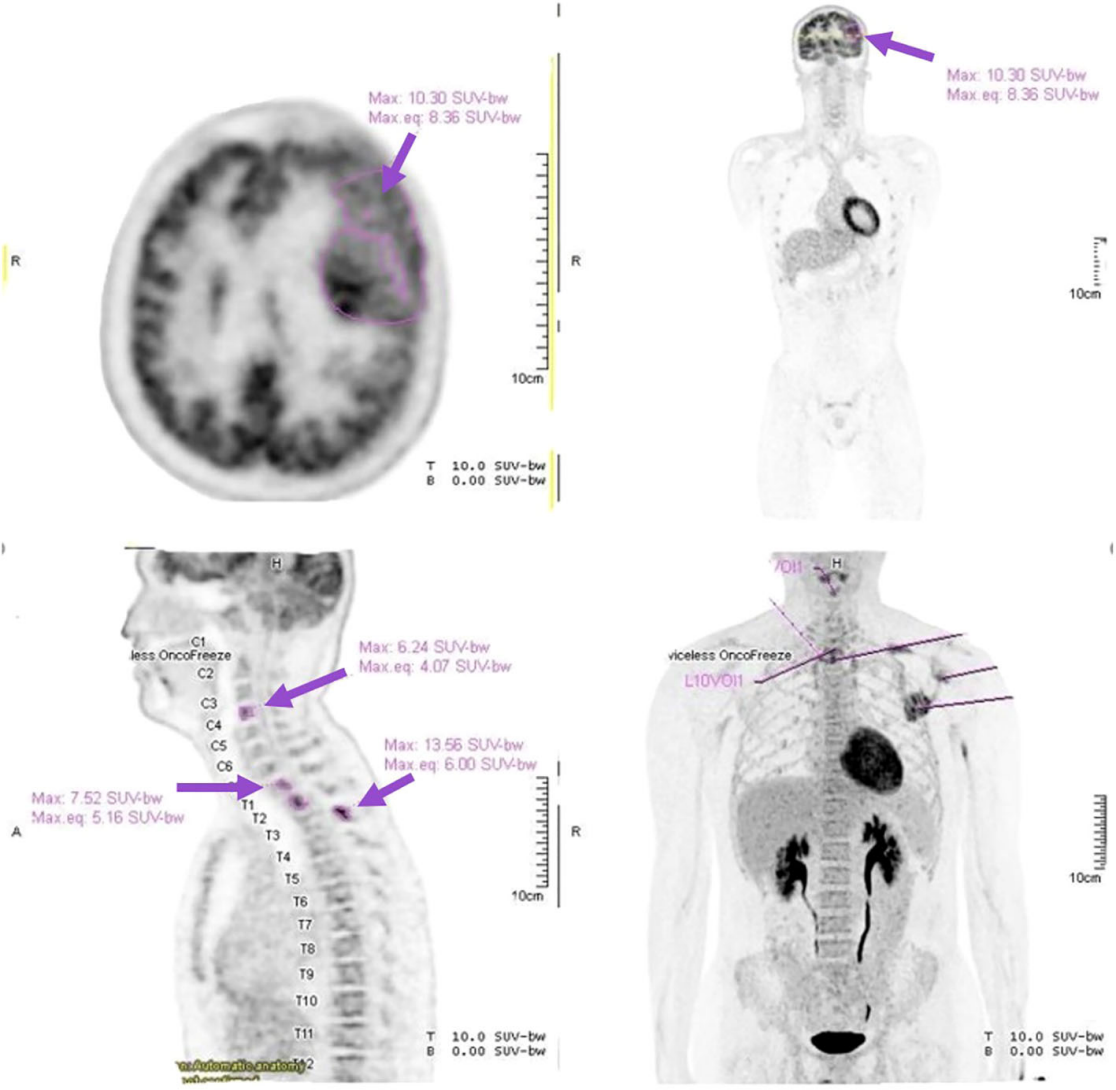
Positron emission tomography (PET). PET imaging demonstrated extensive disease involvement. Axial image revealed intense Fluorodeoxyglucose (FDG) uptake within the left sphenoid–frontotemporal extra-axial mass (purple arrow: Maximum Standardized Uptake Value: 10.3), consistent with high metabolic activity. Sagittal images showed FDG-avid lesions in the cervical and thoracic vertebrae (C4, T1–3) (purple arrows), indicating osseous involvement. Whole-body PET maximum intensity projection image highlighted additional FDG-avid lesions at the left scapula and vertebral levels (purple arrows), confirming systemic dissemination.

High-dose methylprednisolone was continued as pre-phase cytoreductive treatment. Following initiation, the patient’s diplopia, headache, and dizziness improved significantly. Further staging with positron emission tomography revealed metabolically active disease in the left lateral sphenoid with extension to the frontotemporal and orbital regions, as well as skeletal involvement at C4 and T1–3 vertebrae and the left scapula (**Figure 3**). Cytogenetic analysis of bone marrow confirmed the presence of the Philadelphia chromosome, while real-time RT-PCR detected BCR-ABL1 fusion transcripts.

After stabilization of the intracranial mass effect, lumbar puncture was performed and demonstrated an opening pressure of 34 cmH_2_O, confirming elevated intracranial pressure. The patient was subsequently started on systemic therapy with dasatinib in combination with the HyperCVAD chemotherapy regimen. During early induction, he experienced mild transient nausea without vomiting.

Follow-up brain imaging demonstrated marked regression of the intracranial mass involving the left sphenoid wing, dura, and orbit. Repeat bone marrow examination showed minimal residual disease of 0.011% by flow cytometry, and BCR-ABL1 transcript levels decreased significantly compared to baseline. Intrathecal chemotherapy with rituximab, methotrexate, cytarabine, and hydrocortisone was administered as central nervous system–directed therapy. At the latest follow-up, the patient reported no residual visual disturbance. He maintained good functional status and continued systemic treatment with ongoing evaluation for hematopoietic stem cell transplantation.

## Discussion

B-lymphoblastic lymphoma (B-LBL) is a rare and aggressive malignancy of immature B cells that often manifests as extranodal disease in the skin, bone, or mediastinum. Involvement of the central nervous system (CNS) at initial presentation is particularly uncommon and is usually encountered during advanced disease or relapse rather than at onset (1, 3). When present, CNS disease may appear as leptomeningeal infiltration or as parenchymal and extra-axial masses, which can produce elevated intracranial pressure (ICP) and secondary ophthalmic signs. Such findings, ranging from headache and nausea to papilledema, diplopia, or other cranial nerve deficits, can mimic more common neurological or ophthalmological disorders, thereby delaying appropriate diagnosis (4–6).

In B-LBL and precursor B-ALL, CNS involvement at initial diagnosis occurs in approximately 5–10% of adult cases, with CNS-restricted disease (extranodal presentation limited to the CNS) being exceedingly rare (12). Mass lesions resembling brain tumors are even less frequently reported, mostly as sporadic case reports (1, 12). In this patient, a large extra-axial mass produced significant mass effect with radiologic evidence of brain herniation. The documented opening pressure of 34 cmH_2_O further substantiates the diagnosis of true papilledema rather than a compressive optic neuropathy or Foster–Kennedy–like mechanism. Although unilateral proptosis and partial third nerve involvement could suggest a localized compressive process, the presence of bilateral disc edema together with objectively elevated ICP supports a mass effect–driven intracranial hypertension as the principal mechanism.

Additionally, the mild bilateral elevation of intraocular pressure observed at presentation may be secondary to increased episcleral venous pressure associated with intracranial hypertension. This mechanism offers a more plausible explanation than a primary orbital vascular disorder such as carotid–cavernous fistula, particularly in the absence of chemosis, bruit, arterialized conjunctival vessels, or asymmetric orbital congestion.

Our case highlights the critical importance of early fundus examination in patients with headache or ocular symptoms, especially when optic disc swelling is present. The combination of headache, proptosis of one eye, and bilateral disc edema should raise immediate concern for intracranial pathology, including rare causes like hematologic malignancies. Timely ophthalmic evaluation, including fundoscopy and imaging, could enable earlier detection and biopsy, potentially before extensive disease progression.

Several prior reports demonstrate how B-LBL/ALL can mimic orbital or neuro-ophthalmic disorders but seldom include bilateral disc edema as an initial presentation. For example, Ejstrup et al. described extraocular muscle involvement with diplopia (4), Jange et al. reported optic nerve encasement with proptosis (5), and Alsalem et al. showed orbital soft tissue swelling at onset (6). Beyond B-LBL, precursor B-cell acute lymphoblastic leukemia (B-ALL) can also present with ocular signs. Burshina et al. documented a 29-year-old man presenting with diplopia as the first manifestation of B-ALL (7), while Kollia et al. reported bilateral proptosis and papilledema in a pediatric patient later diagnosed with acute lymphoblastic leukemia (ALL) (8). Optic nerve infiltration in relapsed B-ALL has also been associated with papilledema, central retinal vein occlusion, and severe vision loss (9). Additionally, aggressive lymphomas such as diffuse large B-cell lymphoma have been reported to extend to the cavernous sinus, causing diplopia and cranial neuropathies (10, 11).

Taken together, this case emphasizes the need to consider hematologic malignancies in the differential diagnosis of unexplained increased ICP signs, particularly diplopia and optic disc edema. Early neuroimaging and prompt biopsy, coordinated across ophthalmology, neurology and hematology, are essential for accurate diagnosis and timely initiation of therapy.

## Conclusion

This case highlights the rarity of B-lymphoblastic lymphoma (B-LBL) initially manifesting as bilateral optic disc edema due to intracranial mass effect and raised intracranial pressure. The patient’s early symptoms of headache, proptosis, and bilateral optic disc swelling with diplopia served as sentinel clues that mimicked a primary brain tumor. Importantly, earlier fundoscopic examination might have expedited recognition of the underlying pathology. Unlike the more common orbital B-LBL presentations, this case underscores the need for ophthalmologists and neurologists to consider hematologic malignancies in the differential diagnosis of optic disc swelling. Prompt imaging, timely biopsy, and multidisciplinary management are essential for accurate diagnosis and early treatment.

## Data Availability

The original contributions presented in the study are included in the article/supplementary material. Further inquiries can be directed to the corresponding author.
